# Development and psychometric test of core competency assessment scale for gastrointestinal endoscopy nurses

**DOI:** 10.1097/MD.0000000000044969

**Published:** 2025-10-03

**Authors:** Guiqiong Xie, Zhi Zeng, Yazhi He, Xiang Liao, Chaoxiang You

**Affiliations:** a Department of Gastroenterology, Deyang People’s Hospital, Deyang, Sichuan, China; b Hepatobiliary Surgery, Deyang People’s Hospital, Deyang, Sichuan, China.

**Keywords:** core competence, gastrointestinal endoscopy, reliability, scale, specialist nurse, validity

## Abstract

Digestive endoscopy nurses play a pivotal role in contemporary medical settings marked by advancing endoscopic technologies and increasing complexity. Nonetheless, there exists a deficiency in tools tailored for the effective evaluation of their essential competencies. Thus, there is an imperative to develop, validate, and evaluate the reliability and validity of a dedicated assessment scale for this purpose. Guided by role theory and core competency principles, we constructed a scale through literature analysis, semi-structured interviews, and expert consultations. An initial survey involving 413 digestive endoscopy nurses was conducted for item analysis and selection to refine the scale. Subsequently, the scale’s reliability and validity were assessed through a second survey involving 512 nurses, ensuring its robustness and applicability in clinical settings. The developed Core Competency Assessment Scale for Digestive Endoscopy Nurses consists of 41 items organized into 5 dimensions: Professional Attitude, Theoretical Knowledge, Practical Skills, Communication and Coordination Abilities, and Professional Development. Each item demonstrates strong discriminative validity. The cumulative variance explained by these factors is 77.59%. The scale exhibits a Cronbach α coefficient of 0.84 and a test–retest reliability coefficient of 0.80. The content validity index at the item level ranges from 0.80 to 1, and at the scale level, it is 0.96. The Core Competency Assessment Scale for Digestive Endoscopy Nurses demonstrates robust reliability and validity, thereby establishing its suitability for evaluating their essential capabilities in clinical practice.

## 1. Introduction

Endoscopic technology in the digestive system is characterized by its high level of professionalism and invasiveness, making it a crucial method for diagnosing and treating gastrointestinal disorders.^[1]^ With the increasing demand for endoscopic diagnostics and treatments, the team of endoscopy nurses has also grown, evolving into a distinct nursing specialty.^[2]^ The role of nurses has transitioned from merely assisting to becoming collaborators, playing a significant role in the endoscopic treatment process. Research has shown that nurse involvement in colonoscopy can significantly improve the detection rates of polyps and adenomas, and gastroenterology nurses with specialized training can enhance the safety of emergency procedures.^[3,4]^ As endoscopic technology and equipment continue to advance, there is a growing emphasis on the core competencies and specialized care required for gastroenterology endoscopy nurses.^[5]^

The gastrointestinal endoscopy nurse has several important responsibilities that contribute to patient care and procedural efficiency. They assist gastroenterologists during procedures such as gastroscopy and colonoscopy, while also being responsible for cleaning, disinfecting, and maintaining the necessary instruments. One of their key duties is to educate patients about their health and the specifics of the procedure. They also verify patient identity and the purpose of the examination, take medical histories, assist patients in achieving the appropriate position, and provide psychological support to help alleviate anxiety. During the procedures, the nurse monitors patients’ vital signs, assists the physician with endoscopic tasks, and aids in the collection of pathological specimens to ensure patient safety. They skillfully handle instruments during endoscopic surgeries, adjust parameters as needed, collaborate closely with the physician to complete the procedure, and continuously monitor the patient’s condition to respond promptly to any changes. After the examination, they explain post-examination care instructions to patients and their families, issue reports, and organize the examination unit while following protocols for the final cleaning, disinfection, or sterilization of endoscopes, ensuring proper storage and organization of instrument supplies.

Although existing studies have explored training programs for endoscopy nurses, there is still a lack of standardized evaluation tools and criteria.^[6]^ Current efforts have primarily focused on developing tools for assessing endoscopy nurses’ skills but have yet to establish a comprehensive evaluation system. In major gastroenterology endoscopy centers in developed provinces such as Zhejiang, Chongqing, and Shanghai, specialized training programs for gastroenterology endoscopy nurses have been explored and established, with research increasingly focusing on the development of this group. Core competencies are essential for the qualification and recertification of specialty nurses.^[7]^ However, the absence of a validated and standardized tool for assessing these core competencies remains a critical limitation. This deficiency not only hinders consistency in domestic clinical training and evaluation but also underscores the global need for adaptable competency assessment frameworks in specialized nursing fields.

In addition to addressing domestic clinical needs, the development of the Core Competency Assessment Scale for Digestive Endoscopy Nurses also holds promise for international application. The competency domains included in the scale, such as professional knowledge, clinical skills, communication ability, and professional development, are consistent with widely accepted international nursing competency frameworks. These include the standards proposed by the International Council of Nurses and the Canadian Nurses Association.^[8–10]^ The global trend toward standardized assessment in specialized nursing highlights the importance of tools that can be adapted across healthcare systems. Although this scale was developed within the context of Chinese clinical practice, its structure and theoretical foundation offer a basis for cross-cultural adaptation and validation in diverse settings.

Therefore, this study aims to develop a core competency assessment scale for gastroenterology endoscopy nurses and validate its reliability and validity. The goal of this research is to provide a reliable tool for assessing the core competencies of gastroenterology endoscopy nurses, which can support their professional training and performance evaluation. By applying this tool, it is expected to establish a more systematic and standardized evaluation system, thereby advancing the quality of specialized nursing care.

## 2. Materials and methods

### 2.1. Establishment of the research team and the study’s theoretical framework

The research team consisted of 8 members, including a nursing education expert (Master’s thesis advisor), a statistics specialist, a gastroenterology nursing management expert, a gastroenterology medical specialist, and key endoscopy nursing personnel. The team was responsible for literature review and selection, conducting semi-structured interviews, selecting and consulting with experts, distributing survey questionnaires, ensuring quality control, and conducting data analysis.

This study was guided by core competency theory and role theory. Core competency theory, originally proposed by a professor from the University of Michigan Business School in 1990, initially applied to business management, encompasses knowledge, skills, and abilities.^[11]^ Role theory, which evolved from social psychology, defines role as patterned behaviors according to social expectations and specific social statuses.^[12]^ The research team constructed a framework for the core competencies of gastroenterology endoscopy nurses.^[13]^ This framework comprises 5 dimensions: Professional Attitude, Professional Practice Skills, Professional Theoretical Knowledge, Communication and Coordination Abilities, and Professional Development. These dimensions were derived based on the roles and functional requirements of gastroenterology endoscopy nurses.

### 2.2. Establishment of the initial item pool for the scale

**Figure 1.**
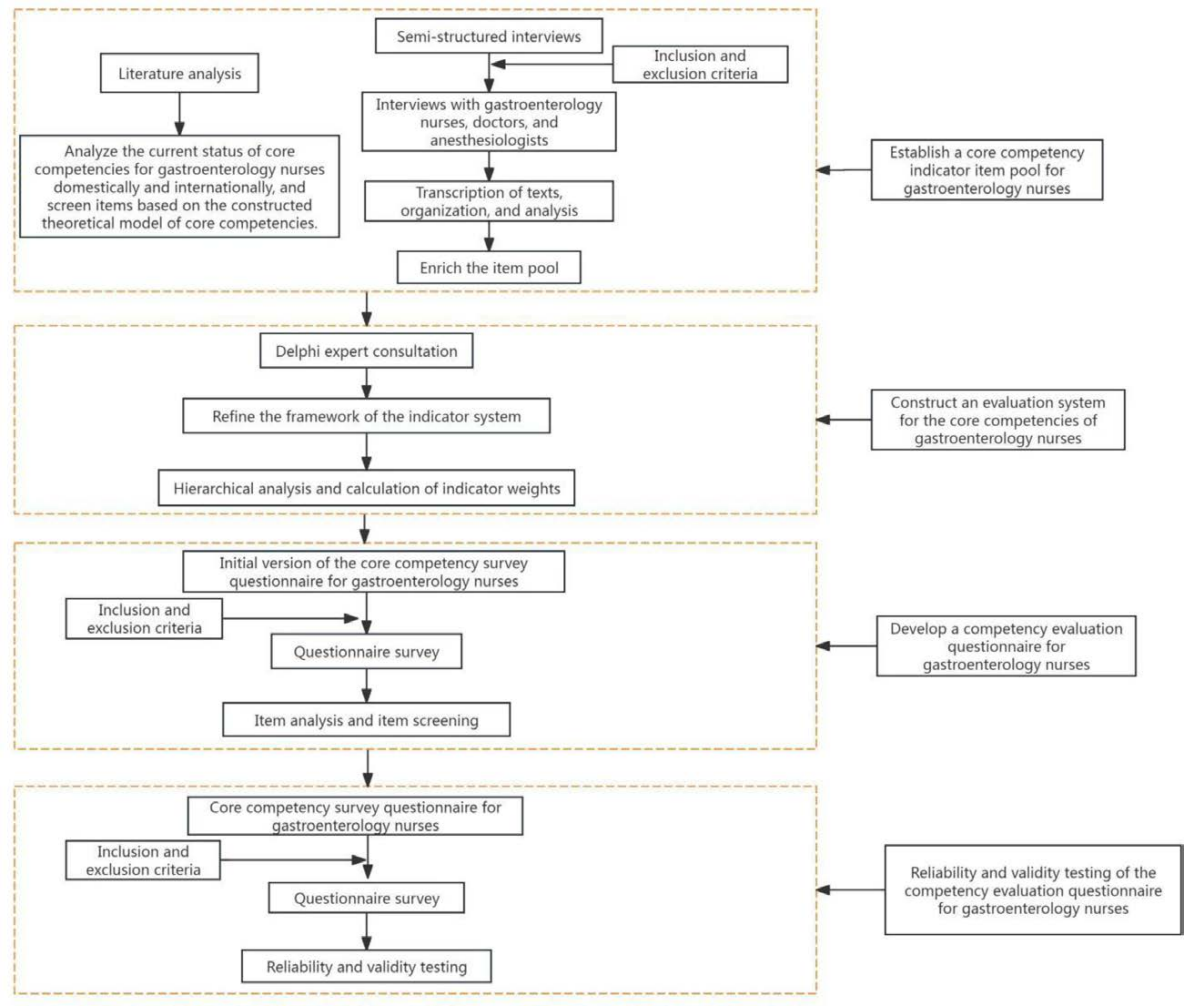
Flow chart of the whole study.

The flowchart of the whole process is shown in Figure 1. From the inception of the self-built database until March 22, 2023, literature researches were searched using keywords such as “gastroenterology endoscopy,” “nurses,” “core competencies,” and “evaluation” in Chinese and English databases including CNKI, Wanfang Data, VIP Database, Chinese Biomedical Literature Database, Chao Xing Journals, as well as PubMed, EMbase, Web of Science, and Cochrane Library. By reading titles, abstracts, and full texts, relevant articles were gradually screened and analyzed to initially select items related to this study, which were then categorized based on the core competency framework for gastroenterology endoscopy nurses. Initially, 729 articles were retrieved, and after eliminating duplicates, inaccessible full texts, and articles unrelated to the research topic, 20 articles were finally included, consisting of 2 qualitative studies, 6 index system constructions, 3 observational studies, 4 quasi-experimental studies, and 5 reviews. Through literature study, an item pool with 5 dimensions and 38 items was initially constructed based on the core competency framework for gastroenterology endoscopy nurses.

Before conducting formal interviews, a semi-structured interview guide was refined based on the research purpose, literature review, group discussion results, and feedback from 2 preinterview subjects. The interview content included the following questions: Please talk about the role of gastroenterology endoscopy nurses in their work? What are the main responsibilities of this role? What abilities do you think gastroenterology endoscopy nurses should have? What is the most important among these abilities? What are the differences between gastroenterology endoscopy nurses and clinical nurses? Which aspects of nurses’ work abilities do you hope to further improve? What are your additional suggestions for improving the core competencies of gastroenterology endoscopy nurses?

During the formal interviews, a general information questionnaire was first distributed, and the purpose and significance of the interview were carefully introduced to obtain informed consent from the interviewees. The interview location was ensured to be relatively quiet and undisturbed, with the entire process recorded and necessary notes taken. Each interview lasted between 30 and 40 minutes.

After the interviews, the recorded data was organized and analyzed using template analysis combined with content analysis, extracting 5 themes and 17 subthemes. Following group discussion, a preliminary evaluation framework for the core competencies of gastroenterology endoscopy nurses was constructed, including 5 dimensions: professional attitude, professional knowledge, professional practice skills, communication and coordination abilities, and professional development abilities, with a total of 44 specific items.

### 2.3. Delphi expert consultation

The criteria for selecting experts include the following points: holding at least a bachelor’s degree and a senior professional title; having over 10 years of relevant work experience in gastroenterology nursing management, nursing education, or endoscopic diagnosis and treatment; voluntary participation in the study. Exclusion criteria include: experts who withdraw from the study midway or fail to complete the questionnaire; individuals with conflicts of interest regarding the expert topic. Based on the initial item pool already established, the research team compiled a consultation questionnaire consisting of 3 parts: background, purpose of the study, and instructions for filling out the questionnaire; general information about the expert, familiarity with the items, and criteria for judgment survey; rating of the importance of core competency items for gastroenterology endoscopy nurses. To ensure questionnaire quality, experts displaying low enthusiasm or with an authority coefficient below 0.70 or poor completion quality will be excluded. The questionnaire will be sent via email to experts with a request to complete it within 2 weeks. Experts are required to self-assess their criteria for judgment and familiarity with the indicators. The importance of each indicator will be assessed using a 5-point Likert scale ranging from “not important” to “very important,” with space provided for expert opinions. Criteria for indicator screening include deletion if the average importance score is below 3.5, the coefficient of variation (CV) exceeds 0.25, or the full score rate is below 40%. Additionally, based on comprehensive expert opinions, dimensions and items of the scale may be added, deleted, merged, or modified. Following 2 rounds of expert consultation, the research team integrated expert opinions to merge, delete, modify, and adjust items, ultimately forming a preliminary scale comprising 5 dimensions and 44 items.

### 2.4. Weighting and assignment of scale items

To determine the weights of primary indicators, this study utilized the Analytic Hierarchy Process through the Statistical Package for the Social Sciences AU online statistical analysis platform to calculate the weights of each item. The weights of secondary indicators were determined by experts based on their risk assessments (on a 5-point scale). The calculation of weights for combinations of secondary items used the product of the weights of primary items (in corresponding proportions) and the weights of secondary items, with rounding based on the combined weights.

### 2.5. Survey subjects

Convenience sampling was used to survey nurses from tertiary hospitals’ gastroenterology endoscopy centers via the ``Wenjuanxing’’ platform. Inclusion criteria: full-time nurses working in gastroenterology endoscopy centers; voluntary participation in the study. Exclusion criteria: nurses with <6 months of experience in rotational or fixed positions in gastroenterology endoscopy centers; nurses in training, internships, or residency programs. Considering the principle that the sample size for survey studies is generally 5 to 10 times the number of questionnaire items,^[11]^ and accounting for a 10% attrition rate, the determined sample size is: 248 to 495 for item analysis surveys, and 226 to 451 for validity and reliability testing surveys.

### 2.6. Data collection and quality control methods

Each returned questionnaire was reviewed individually to eliminate invalid responses such as those with identical answers for each option or obvious contradictions. Data were exported from the ``Wenjuanxing’’ platform. The questionnaire’s first page included an informed consent form, survey purpose, and instructions for completion. Each ID was allowed only one response, which could be submitted only after completing all questions. Questionnaires with excessively short completion times, irregular response patterns, or inconsistent answers were excluded.

### 2.7. Validity analysis

Validity analysis in this study involves assessing the extent to which the questionnaire measures what it intends to measure.^[12]^ First, this study calculates the correlation coefficient between each item and the total score of the questionnaire. Items with a correlation coefficient < 0.5 are considered for deletion, as they may not adequately correlate with the overall construct being measured. Second, we used critical ratio method, items are divided into high and low scoring groups based on the top and bottom 27% of total scores. A *t* test is then conducted between these groups. Items with a *t* statistic > 3 and *P* < .05 are retained, indicating significant differences between high and low scoring groups. Third, Cronbach α coefficient is calculated for each dimension initially.^[14]^ Then, for each item, Cronbach α is recalculated after systematically removing that item. If removing an item leads to an increase in the Cronbach α coefficient for its dimension, the item may be considered for deletion, as it suggests that the item does not contribute to the internal consistency of the scale. Finally, factor analysis is used to examine the underlying structure of the questionnaire. Items with factor loadings < 0.45 or items loading onto multiple factors with similar loadings and no significant differences may be considered for deletion. Before conducting factor analysis, the suitability is assessed through Kaiser–Meyer–Olkin (KMO) measure and Bartlett test of sphericity.^[13]^ KMO value > 0.7 and a significant Bartlett test (*P* < .05) indicate that factor analysis is appropriate for the data.^[15]^ Furthermore, content validity is assessed by consulting experts who rate the importance of each item on the scale. This process calculates the Item-Level Content Validity Index (I-CVI) and the Scale-Level Content Validity Index (S-CVI). An I-CVI > 0.70 and S-CVI > 0.80 indicate good content validity for both individual items and the overall scale.^[16]^ Principal component analysis with maximum variance rotation is used to explore the data. Factors are extracted based on eigenvalues ≥ 1, cumulative variance contribution exceeding 50%, and each factor containing at least 3 items. Items with factor loadings ≤ 0.400 or with loadings ≥ 0.400 on multiple factors with a difference < 0.150 may be considered for deletion.

### 2.8. Reliability analysis

Cronbach α coefficient is used to assess internal consistency. A coefficient > 0.70 is generally acceptable, while values between 0.80 and 0.90 indicate good internal consistency.^[17]^ Values exceeding 0.90 indicate very high internal consistency. To assess test–retest reliability, 50 survey participants are conveniently selected and surveyed again after a 2-week interval. A correlation coefficient > 0.7 indicates good stability of the scale over time.

### 2.9. Statistical methods

All data were entered into Excel following double-checking procedures and analyzed using SPSS 26.0 software. Descriptive statistics utilized mean ± standard deviation for continuous variables and frequency with percentages for categorical variables. Expert consultation results were evaluated using indices such as positivity coefficient, authority coefficient, variation coefficient, Kendall coefficient of concordance, among others. Survey data from the pilot test of the questionnaire underwent item analysis and assessments of reliability and validity, with statistical significance set at *P* < .05.

## 3. Results

### 3.1. Inclusion of expert information

The study ultimately included 15 experts from 10 tertiary general hospitals across 6 provinces and municipalities: Sichuan, Shanghai, Zhejiang, Chongqing, Guizhou, and Lanzhou. The panel comprised nursing management, education specialists, and endoscopy medical experts who completed 2 rounds of consultation. Of the included experts, 6 were male and 9 were female, with an average age of 39.2 ± 9.2 years. Their average tenure in digestive endoscopy centers was 17.2 ± 7.78 years. The panel consisted of 9 experts with bachelor’s degrees and 6 with master’s degrees. In terms of professional titles, 8 held associate senior positions and 7 held full senior positions.

### 3.2. Expert panel consultation

The study conducted 2 rounds of expert consultations. In the first round, the positivity coefficient was 93.75%,^[18,19]^ with a criteria adoption (Ca) of 0.87, familiarity (Cs) of 0.77, and authority coefficient (Cr) of 0.82. The CV ranged from 0.00 to 0.23. Based on item selection principles and comprehensive expert opinions through group discussions, 4 items were added, 3 were deleted, 2 were split, 1 was merged, and descriptions of 3 items were adjusted. The revised items formed the basis for the second round of consultations with the same group of experts. In the second round, the positivity coefficient was 100%, with CV ranging from 0.00 to 0.21, and only 1 expert suggested a modification related to sentence structure.

Kendall W coefficients for primary, secondary, and tertiary indicators in the first round were 0.161, 0.352, and 0.141, respectively, and in the second round were 0.292, 0.352, and 0.155. Following both rounds of expert consultations, a core competency evaluation system for digestive endoscopy nurses was established, encompassing 5 dimensions: professional attitude, theoretical knowledge, practical skills, communication and coordination, and professional development capability, consisting of 45 items.

### 3.3. Item analysis results

Correlation coefficient analysis indicated correlations between item scores and total questionnaire scores ranging from 0.54 to 0.82 (*P* < .01), demonstrating significant positive correlations between each item and the overall questionnaire score. No items were deleted based on this analysis. Analysis using the critical ratio method revealed statistically significant differences between high and low scoring groups for all items (*P* < .001), with all critical ratio values exceeding 10, leading to retention of all items.

Internal consistency testing using Cronbach α coefficients for the questionnaire’s 5 dimensions yielded values of 0.798, 0.946, 0.959, 0.952, and 0.961, respectively. Removal of item 32 resulted in improved Cronbach α values for its corresponding dimension, leading to its deletion. Results from the KMO measure of sampling adequacy were 0.786, and Bartlett test of sphericity showed a significant value of 31,263.877 (*P* < .001), confirming suitability for factor analysis.

Exploratory factor analysis (EFA) using principal component analysis and maximum variance rotation in the first round identified items 37 and 40 loading on 2 common factors with loadings below 0.45. These items were removed, and a second round of factor analysis on the remaining items identified item 27 with loadings below 0.45 across all factors, prompting its removal. In the third round of EFA, all items showed loadings between 0.457 and 0.806 on their respective factors, all exceeding the threshold of 0.45. Following item analysis, 41 items were retained for the final questionnaire.

### 3.4. Reliability analysis results

The overall Cronbach α coefficient for the scale was 0.841, with individual dimensions ranging from 0.798 to 0.959. A follow-up survey conducted 2 weeks after the initial survey with 50 nurses who had completed the questionnaire showed Pearson correlation coefficients ranging from 0.718 to 0.835 across dimensions. The test–retest reliability of the scale was 0.801 (*P* < .001) (Table 1).

**Table 1:** Results of questionnaire reliability test.

Items	Cronbach α	Retest reliability	*P*
Professional attitude	0.798	0.718	<.001**
Professional theoretical knowledge	0.947	0.795	<.001**
Professional practice ability	0.959	0.835	<.001**
Communication and coordination ability	0.954	0.829	<.001**
Professional development capability	0.954	0.825	<.001**
Total schedule	0.841	0.801	<.001**

**P <.01.

### 3.5. Validity analysis results

Content validity was assessed using I-CVI, which ranged from 0.80 to 1.00, all exceeding the recommended threshold of 0.78. The S-CVI, computed as 0.96, also surpassed the minimum criterion of 0.90, indicating excellent content validity for the scale.

Prior to the main study, a pilot survey involving 10 nurses confirmed that the questionnaire items were easily understandable without ambiguity, demonstrating good face validity.

EFA was conducted to evaluate construct validity. The Kaiser–Meyer–Olkin measure was 0.974, indicating sampling adequacy, and Bartlett test of sphericity was highly significant (*χ*² = 13,801.164, *P* < .001), confirming the suitability of the data for factor analysis.

Using principal component analysis with maximum variance orthogonal rotation, 5 factors with eigenvalues >1 were extracted, consistent with the initial model. These factors accounted for a cumulative variance of 77.587%. Factor names and loadings are detailed in Table 2, confirming the construct validity of the Digestive Endoscopy Nurse Core Competency Assessment Scale.

**Table 2:** Factor naming and loading values of the core competency assessment scale for digestive endoscopy nurses.

Factor	Item	Loading values
1	Professional attitude	
	1. I love endoscopic nursing, have a high sense of responsibility and self-dedication.	0.687
	2. Be disciplined and strictly abide by the standard procedures and operating norms.	0.799
	3. Have proactive service awareness and good team spirit.	0.662
2	Professional theoretical knowledge	
	1. Master the anatomy, physiology and pathophysiology of digestive tract.	0.660
	2. Familiar with digestive system disease diagnosis process, treatment and common gastrointestinal disease care expertise.	0.788
	3. Familiar with the common drugs and pharmacology of digestive diseases.	0.736
	4. Master the technical specifications of digestive endoscope cleaning and disinfection.	0.689
	5. Master the knowledge of disinfection and sterilization effect monitoring of endoscope center and nosocomial infection prevention and control.	0.735
	6. Master the construction of various digestive endoscopes and instruments, and the use and maintenance of instruments and equipment.	0.735
	7. Master the indications, contraindications, prevention and treatment of related complications of endoscopic diagnosis and treatment.	0.815
	8. Master the core steps of various digestive endoscopic diagnosis and endoscopic surgical treatment.	0.737
	9. Master the preoperative preparation, intraoperative cooperation and postoperative nursing knowledge of various digestive endoscopy and endoscopic surgery.	0.766
	10. Knowledge of occupational safety and protection.	0.724
	11. Knowledge of emergency rescue.	0.754
	12. Knowledge of law and ethics.	0.694
3	Professional practice ability	
	1. Master the skills of cleaning and disinfecting digestive endoscopes.	0.692
	2. Have the ability of biological monitoring and quality control of endoscopes, surgical instruments and diagnosis and treatment environment.	0.734
	3. Master the use of various instruments, instruments, equipment and articles in endoscopy center.	0.775
	4. Have the ability to maintain and maintain all kinds of endoscopes, accessories, instruments and instruments.	0.702
	5. It can accurately evaluate the needs of patients and their families in the diagnosis and treatment of digestive endoscopy.	0.803
	6. Ability to perform risk assessment on patients undergoing endoscopic treatment.	0.805
	7. Have the ability to complete various diagnostic digestive endoscopy nursing cooperation.	0.716
	8. Have the ability to complete all kinds of therapeutic digestive endoscopy nursing cooperation.	0.812
	9. Ability to cooperate in the management of common complications in digestive endoscopy and endoscopic surgery.	0.717
	10. Have the ability to standardize the treatment of specimens.	0.711
	11. Have the ability to observe changes in the condition of patients undergoing endoscopic diagnosis and treatment.	0.699
	12. Have emergency treatment skills for patients with mandibular loss, bed fall, fall and other nursing risk events.	0.772
	13. Have first aid skills of cardiopulmonary resuscitation and electrical defibrillation.	0.656
4	Communication and coordination ability	
	1. Ability to communicate effectively with medical staff during digestive endoscopy diagnosis and treatment.	0.804
	2. Have the ability to effectively communicate with patients and family members during the diagnosis and treatment of digestive endoscopy.	0.843
	3. Have the ability to coordinate the daily work order of endoscopy center.	0.758
	4. Have the ability to educate patients and their families on specialized knowledge and health guidance.	0.793
5	Professional development capability	
	1. Have a sense of active learning and pay attention to new developments in the field of specialization.	0.788
	2. It can learn new knowledge, new theories and new techniques in the field of digestive endoscopy through various ways.	0.787
	3. Ability to teach theoretically.	0.809
	4. Ability to manage costs and use resources efficiently.	0.838
	5. It can evaluate the quality and safety of endoscopic nursing work, and put forward continuous improvement measures.	0.792
	6. It can carry out follow-up management for patients treated with digestive endoscopy.	0.698
	7. Able to search domestic and foreign literature and write scientific research papers.	0.598
	8. Have the ability of invention and innovation.	0.650
	9. Fund or project declaration, scientific solution to clinical problems.	0.685

## 4. Discussion

### 4.1. The Core Competency Assessment Scale for Digestive Endoscopy Nurses demonstrates scientific rigor

The development of measurement scales is essential in clinical practice, as adherence to rigorous procedures is critical for ensuring their scientific validity and reliability.^[20]^ This study aimed to create a core competency assessment scale for gastrointestinal endoscopy nurses by following a systematic process to guarantee accuracy and dependability.

The scale was grounded in core competency theory and role theory.^[11,12]^ Core competency theory emphasizes the essential skills and knowledge required for nurses, while role theory delineates the roles and responsibilities of nurses in clinical settings. The initial item pool, developed through a literature review and semi-structured interviews, reflects the current state of nursing practice in China and addresses specific clinical needs. This alignment ensured that the scale’s items are both relevant and applicable.

During the expert review phase, 15 experts from major tertiary hospital gastrointestinal endoscopy centers, including nursing managers, educators, and medical specialists with over 10 years of experience, evaluated the scale. These results align with previous studies highlighting the importance of expert consensus in developing reliable assessment tools.^[21–24]^ Additionally, item-by-item analysis and subsequent refinement are critical steps in the development of a high-quality measurement scale.^[24]^ Our study employed comprehensive statistical analyses (including correlation coefficient analysis, critical value analysis, homogeneity testing, and factor analysis) which provided more robust validation of the scale items.

### 4.2. The Core Competency Assessment Scale for Digestive Endoscopy Nurses demonstrates good reliability and validity

Reliability is a critical indicator for evaluating the accuracy and consistency of a measurement scale.^[25]^ Our findings indicate that the scale demonstrates high reliability and stability. This consistency suggests that the scale is capable of providing reliable measurement results across different time points and samples, affirming its robustness and dependability.

Validity is crucial for assessing whether the measurement tool accurately captures the intended attribute.^[26]^ Content validity evaluates how well the scale’s content reflects the intended domain.^[25]^ Our study has demonstrated strong content validity. Furthermore, EFA revealed 5 factors with eigenvalues >1, which aligns with the initial model, and the cumulative variance contribution rate was 77.587%. All items had factor loadings >0.45, indicating robust structural validity.

The high Cronbach α and test–retest reliability values in this study highlight the internal consistency and stability of the scale, which are consistent with findings from similar studies.^[27,28]^ These results suggest that the Core Competency Assessment Scale performs reliably in measuring the core competencies of gastrointestinal endoscopy nurses.The reliability metrics align with the standard benchmarks in the field, demonstrating that our scale is a dependable tool for evaluating nursing competencies.

In terms of validity, our content validity results are comparable to those found in other validated scales in healthcare settings.^[26,29]^ The I-CVI and S-CVI values indicate that the scale’s content is well-aligned with the core competencies expected for gastrointestinal endoscopy nurses. The EFA supports the scale’s construct validity, showing a clear factor structure that mirrors theoretical expectations, similar to findings in other validated instruments.^[30]^

Additionally, a pilot survey prior to the main study, involving 10 nurses confirmed that the questionnaire items were easily understandable without ambiguity, demonstrating good face validity. The pilot study conducted before the main investigation served several important purposes. Firstly, it allowed us to test the feasibility of the core competency assessment scale for endoscopy nurses in a real-world setting, providing preliminary insights into its practicality and usability. During the pilot phase, we were able to identify potential challenges in the assessment process, such as ambiguities in certain items and variations in interpretation among participants. This feedback was invaluable, leading to iterative refinements of the scale to enhance clarity and relevance. Moreover, the pilot study also facilitated initial data collection, which helped us gauge the expected response rates and participant engagement levels, thus informing our approach for the larger study. The findings from the pilot were promising, indicating strong internal consistency and initial validity, thereby reinforcing our confidence in proceeding with the full-scale study.

Compared to other tools in the literature, our scale offers a comprehensive approach to competency assessment, enhancing both the precision and applicability of competency evaluations in gastrointestinal endoscopy nursing.

### 4.3. The Core Competency Assessment Scale for Digestive Endoscopy Nurses exhibits specialized characteristics and practicality

The improvement of digestive endoscopy nursing quality is closely tied to the development of a professional talent team. Digestive endoscopy nurses have evolved into a sizable group of nurses with distinct professional characteristics.^[31]^ The Core Competency Assessment Scale for Digestive Endoscopy Nurses developed in this study demonstrates clear professional relevance and practical utility. This scale evaluates core competencies from 5 dimensions: professional practical skills, theoretical knowledge, communication and coordination abilities, professional development capabilities, and professional attitude. It effectively reflects the specific demands of digestive endoscopy nursing and aligns with current trends in professional development both domestically and internationally.^[32]^

The scale’s design addresses the unique aspects of digestive endoscopy nursing, distinguishing it from more general nursing assessment tools. Compared to existing research, which primarily focuses on training needs, models, and methods (e.g., references,^[33–36]^ this scale provides a comprehensive and precise evaluation of nurses’ competencies in their specialized field). This comprehensive assessment aligns with findings from similar studies that underscore the necessity of specialized evaluation tools in professional settings.

The Core Competency Assessment Scale holds significant implications for the field of digestive endoscopy nursing. It offers a robust tool for nursing managers to gain an in-depth understanding of their staff’s overall competency levels. By identifying strengths and weaknesses, this scale facilitates the development of targeted training and development plans, ultimately enhancing nursing practice and improving overall care quality. Moreover, the scale supports the objective selection of suitable staff and optimizes human resource allocation, contributing to a rapid enhancement in the quality of digestive endoscopy nursing.

It is important to emphasize the advantages of our proposed core competency assessment scale for endoscopy nurses as compared to existing assessment methods. Traditional assessment approaches often rely on subjective evaluations or generic competency checklists that may not fully capture the specific skills and knowledge required in the specialized field of endoscopy nursing. In contrast, our assessment scale is tailored to address the unique competencies critical for effective practice in this specialty. One key advantage of our scale is its evidence-based framework, which integrates the latest clinical guidelines and best practices in endoscopy care. This ensures that the competencies assessed are relevant and aligned with current industry standards, providing a more accurate reflection of a nurse’s readiness to perform in this role. Additionally, our scale incorporates both technical skills and soft skills, such as communication and teamwork, which are essential for ensuring patient safety and enhancing overall care quality. Furthermore, the implementation of our assessment scale encourages a more standardized approach to evaluating nurse competencies across different healthcare settings. This consistency not only aids in identifying areas for improvement but also facilitates targeted professional development initiatives, ultimately leading to improved patient outcomes. Future research should further explore the practical implications of implementing this scale in diverse clinical environments to comprehensively validate its effectiveness.

This study has several limitations that should be considered when interpreting the findings. First, although we made efforts to recruit participants from various institutions, all respondents were drawn from tertiary hospitals in China. This geographic and institutional homogeneity may limit the external validity of the scale, particularly concerning cross-cultural or regional differences in clinical practice. Future studies should incorporate larger, more diverse, and international samples to enhance the generalizability and cultural adaptability of the scale. Second, criterion-related validity testing was not conducted, as there is currently no established and validated external scale specifically designed for gastrointestinal endoscopy nurses in China. The absence of a recognized benchmark limits the ability to evaluate the concurrent validity of our scale. Future research should aim to identify or develop appropriate external measures to further confirm the effectiveness of the tool. Third, although EFA supported the structural validity of the scale, confirmatory factor analysis (CFA) was not performed. To provide stronger support for the theoretical model, future studies should conduct CFA using independent and sufficiently large samples. Finally, while this study identified 5 core competency domains through EFA, it did not explore the structural relationships among these dimensions. The absence of structural equation modeling (SEM) limits the ability to assess the interactions between latent constructs and the theoretical coherence of the scale. Future research should apply SEM to investigate potential mediating or hierarchical relationships among the competency domains. This approach may offer a more comprehensive understanding of the internal structure and integrative function of the competencies assessed.

Compared with other validated competency assessment tools in the nursing field, such as the Core Competency Scale for Extracorporeal Membrane Oxygenation nurses^[7]^and the Nurses’ Disaster Core Competency Scale,^[25]^ our scale shares a similar structural foundation in terms of multidimensionality and theoretical guidance. However, it differs in its domain specificity and item construction. For instance, while the Extracorporeal Membrane Oxygenation scale emphasizes advanced life support techniques and crisis decision-making, our scale is uniquely tailored to reflect the procedural, technical, and patient-centered competencies essential to gastrointestinal endoscopy nursing (such as instrument handling, disinfection protocols, and intraoperative cooperation).

Furthermore, unlike more generalized nursing competency scales, our instrument integrates both technical and soft skills specific to digestive endoscopy practice. This includes not only theoretical knowledge and practical skills, but also professional development and communication coordination competencies, which are often underrepresented in other models. These distinctions suggest that our scale fills an important gap by offering a comprehensive, targeted, and context-sensitive tool for evaluating core competencies in this highly specialized nursing field.

In conclusion, the Core Competency Assessment Scale for Digestive Endoscopy Nurses, developed in this study, consists of 5 dimensions and 41 items. It features a moderate item length, clear language, strong reliability and validity, and structural stability, making it a practical tool for assessing core competencies in this specialized field. Future research should focus on several key areas: conducting CFA with independent and larger samples to verify the scale’s structural validity; applying SEM to explore latent relationships and theoretical pathways among competency domains; developing or identifying appropriate external criterion measures to establish criterion-related validity; and evaluating the scale’s adaptability and performance in cross-cultural and multi-institutional settings. These directions will help further refine the scale and enhance its applicability in guiding professional training, competency evaluation, and quality improvement in digestive endoscopy nursing.

## Acknowledgments

The authors would like to express their gratitude to all participants who accepted this study.

## Author contributions

**Conceptualization:** Zhi Zeng.

**Data curation:** Guiqiong Xie, Yazhi He.

**Formal analysis:** Guiqiong Xie.

**Investigation:** Guiqiong Xie.

**Methodology:** Guiqiong Xie, Xiang Liao, Chaoxiang You.

**Resources:** Zhi Zeng.

**Writing – original draft:** Guiqiong Xie, Yazhi He, Xiang Liao, Chaoxiang You.
